# Mechanical behavior and waterproof performance of longitudinal section of tunnel segment joint gasket

**DOI:** 10.1038/s41598-024-60990-y

**Published:** 2024-05-08

**Authors:** Qian Zhang, Li Xie, Zhirong Zhao, WeiGang Zhao, Yan Ma, Longhe Shi, Zihang Zhang

**Affiliations:** 1 Shijiazhuang Tiedao University, Shijiazhuang, 050043 China; 2 Shuohuang Railway Development Co., Ltd, Suning, 062350 China

**Keywords:** Civil engineering, Engineering

## Abstract

In the construction stage, due to construction errors and longitudinal differential settlement during tunnel operation, the amount of dislocation and opening at the segment joint increases, increasing the likelihood of water leakage. Therefore, it is necessary to conduct an in-depth study on the influence of the amount of dislocation and opening at the segment joints on the contact stress of the longitudinal section. Firstly, through theoretical analysis, this paper deduces that the waterproof performance of the gasket depends not only on its own contact area, linear compression stiffness, and Poisson’s ratio but also on the height of the segment joint specimen and the amount of joint opening caused by the sinking offset angle. Then, the effects of different openings and dislocations at the segment joints on the contact stress of the segment gasket section were compared using numerical simulation and model experiments. Through numerical simulation, it is found that the dislocation has a greater influence on the longitudinal left section. The average contact stress at 16 mm is 28.3% lower than that at 4 mm, and the influence of the opening amount on the sealing gasket section is greater than that of the dislocation. Combined with the test results, it is also shown that the influence of the opening amount of the waterproof performance at the segment joint is greater than that of the dislocation, and the waterproof rate of the segment gasket section joint is greater than 40% under the modified working condition.

## Introduction

The vigorous development of China’s transportation industry and the rapid progress of tunnel construction technology provide a broad space for the development and application of underwater tunnels. Waterproofing is very important for underwater tunnels, especially for tunnels constructed by the shield method. Waterproofing at segment joints is an important part of tunnel waterproofing^1–3^. Under the action of long-term complex geology and high water pressure, joint leakage has become a serious problem that hinders the normal use of tunnels^4^. For shield tunnels subjected to ultra-high water pressure, how to reduce the risk of water leakage in tunnels is becoming a new academic and engineering hotspot^5^.

At present, research on the waterproofing of tunnel segment joints mainly focuses on model testing and numerical analysis. In terms of numerical simulation, Wang et al.^6,7^ used the ABAQUS software to analyze the curves of the compression forces of multiple gaskets under different hardness and obtained the waterproof failure modes of these gaskets under various misalignments. Chen et al.^8^ established a nonlinear finite element model to simulate the behavior of gasket-type integral sealants. The calculation results show that with the increase of joint opening or joint rotation, the waterproof ability of the joint is significantly reduced. Zhang et al.^9^ used the finite element analysis method to study the waterproof effect of gaskets under different segment joint angles. The results show that the piecewise opening angles have a linear positive correlation with the ovality. At the same time, the influence of different side seam opening angles on the waterproof effect of the gasket is also different, which shows that the outer angle is greater than the inner angle. Xue et al.^10^ used ANSYS to establish a three-dimensional finite element model of a gasket and groove and adopted the loading mode of ‘first compression and then staggered joint’ to simulate the waterproof ability of the gasket. They found that after considering the water pressure, the waterproof ability of the gasket ‘first compression and then staggered joint’ was improved compared with that without water pressure. This indicated that the effect of water pressure further compressed the gasket, increasing the surface contact stress and improving the waterproof ability. Gong et al.^11^ used numerical simulation to compare and analyze single-type gaskets and composite gaskets. The research showed that when the joint opening is large, the contact stress is mainly caused by the expansion of the rubber in water. Appropriately increasing the width and thickness of the water-expanded rubber block is beneficial to the secondary waterproofing of a composite gasket. Konrad et al.^12^ found, through a large number of experimental studies and numerical calculations on two kinds of PTFE-filled wound gasket structures, that the asymmetric gasket under load has a larger effective contact surface compared to a standard gasket. In the aspect of the model experiment, Faisal et al.^13^ studied the mechanical behavior of the sealing gasket at the segment lining joint of the subway shield tunnel under static load and seismic load, respectively. The results show that the cracking area of the sealing gasket groove, the contact surface of the sealing gasket and the position of the bolt hole are the main leakage parts. Dong et al.^14^ carried out a water pressure test on a commonly used gasket for segment lining joints, analyzed and deduced the waterproof mechanism of the gasket, and analyzed that the waterproof performance of the gasket is composed of two parts: extrusion sealing resistance and self-sealing resistance. In order to study the shear mechanical response and deformation failure law of F-type socket joint of rectangular pipe jacking tunnel under different base weights, Xu et al.^15^ adopted the method of indoor test and numerical simulation. The results show that the deformation process of shear joints includes four stages: gap closure, elastic expansion, shear strengthening and yield failure. Through the detailed experimental study of the two-component material in the creep stage, Oggeri et al.^16^ think that the creep phenomenon of the two-component material can lead to the reduction of the load applied to the support system and the increase of the deformation of the tunnel wall. Ding et al.^17^ developed a waterproof performance test device for gaskets that can accurately simulate the amount of dislocation and opening and studied the compressive load and contact surface contact stress of several different cross-section gaskets. It was found that the water leakage parts of the gaskets in the test were mainly the joints and corners, and there was a ‘correlation zone’ between the joint waterproof test and the gasket compression test. Based on the stress and deformation characteristics of segment joints in a subway tunnel, Shi et al.^18^ designed and conducted the sealing test of segment joints. They obtained the performance of the segment joint sealant under different joint openings and dislocation deformations. It was concluded that the joint openings and dislocation deformations can significantly decrease the critical pressure of the sealant. Ye et al.^19^ studied the influencing factors of the mechanical properties of EPDM rubber gaskets for shield tunnel segment joints through a self-designed test system. The results showed that the shape of the opening, the opening rate of the section, the amount of dislocation, rubber hardness, and the number of slots were primary and secondary factors influencing the contact stress distribution of the gaskets.

In general, at the present stage, most of the research on the simulation analysis of tunnel segments focuses on the joint gasket section. In waterproof tests, the segment structure is often replaced by a steel plate. However, there have been few studies conducted on the longitudinal analysis of the joint gasket, and the real waterproof performance of tunnel segment joint gaskets has not been thoroughly investigated. Without research conducted in these two aspects during actual projects, it is impossible to truly and comprehensively understand the waterproof performance of tunnel segment joint gaskets. In light of this, this study aims to design a relevant test system and conduct numerical simulations using the thickness and material design of a prototype segment in a subway tunnel in Shenzhen. The influence of different tunnel segment joint openings and dislocation on the contact stress of the longitudinal section of the segment gasket will be investigated. The results of these two analyses will be compared to explore the waterproof performance of tunnel segments, providing a foundation for the practical application of waterproof gaskets in tunnel segment joints.

## Theoretical analysis

### Waterproof model of sealing gasket for segment joint

The waterproof form of the tunnel segment joint gasket selected in this study is the type of separate arrangement of inside and outside, that is, two rubber gaskets are arranged separately on the upper and lower sides of the segment joint section, and the lower side of the rubber gasket is preset in the lower part of the water expansion sealing strip. Seam material protection, waterproof failure on the upper side of the gasket is waterproofed by the lower side, the composite elastic rubber gasket is designed separately from the water expansion sealing strip, and the bolt holes are reserved in the two composite elastic rubber gaskets, so that the waterproof material on the surface of the segment can give full play to the waterproof effect.

The waterproof working principle of the annular gasket at the joint of the segment structure mainly includes the following two aspects:

1. *Extrusion sealing*: Under the action of axial load pressure, the annular gasket produces the axial extrusion pressure of the segment, which improves the sealing effect of the gasket. The sealing performance and lifespan of the sealing gasket are two important indicators of the amount of compression.
2. *Self-sealing effect*: The water pressure acts on the inner wall of the segment joint, and the gasket produces the tensile deformation of the expansion in the gasket, thus increasing the friction and shear force of the axial contact interface and improving the sealing effect.

**Figure 1:**
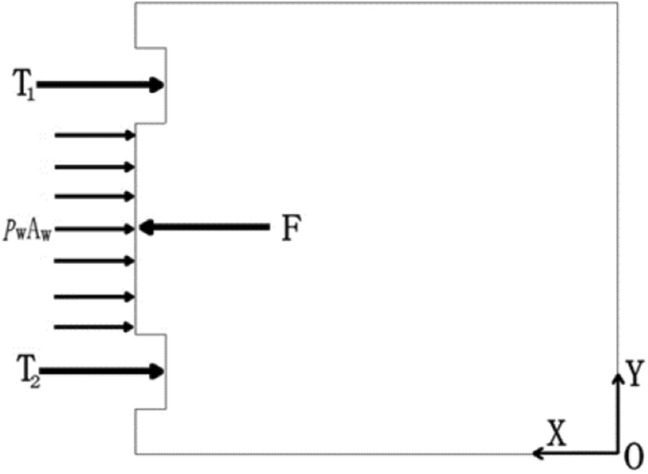
Waterproofing model of segment longitudinal section joint gasket.

When the joint of the tunnel segment structure is subjected to water pressure, the effect of water pressure on the gasket can be decomposed into radial and vertical directions. The radial water pressure has a shear effect on the contact surface of the gasket to overcome the friction resistance among the gaskets. The vertical water pressure reduces the contact pressure stress on the surface of the gasket, creating conditions for the radial water pressure to break through the seal. If the joint structure is staggered, in addition to the above load, it will also generate friction among the gaskets or among the contact surface of the test block. It is assumed that the unit width of the longitudinal section of one side of the two continuous tunnel segments is selected for force analysis, and the waterproof model of the annular sealing gasket of the longitudinal section of the segment is established^20^, as shown in Fig. 1. Among them, Pw represents the magnitude of water pressure, Aw represents the axial water pressure area along the joint, and T represents the elastic restoring force of the gasket. Due to the different openings on the upper and lower sides of the joint of the tunnel segment structure caused by external factors, the elastic restoring force also varies. F represents the friction among the gaskets. In addition to the forces mentioned above, other external forces, such as the connection load of the tunnel segment structure and the selfweight of the structure, are denoted as F.

### Mechanical calculation

It is assumed that the sealing waterstop and specimens conform to the basic assumptions of elastic mechanics. As shown in Fig. 1, at the time of t0, when the joint specimens are not subjected to water pressure, but the sealing waterstop is, the equilibrium state is maintained, and the resultant force in the X direction is zero.

1 $$T_{1} ({\text{t}}_{0} ) + T_{2} ({\text{t}}_{0} ) - F({\text{t}}_{0} ) = 0$$

At time t1, when the assembled joint is subjected to water pressure due to external factors, if the other external force F is unchanged at this time, there is

2 $$T_{1} (t_{1} ) + T_{2} (t_{1} ) - F(t_{1} ) - P_{{\text{w}}} A_{w} = 0$$

From Eqs. (1) and (2), we know

3 $$\Delta T_{1} + \Delta T_{2} = P_{{\text{w}}} A_{{\text{w}}}$$

The compression line of the waterstop belt, with a stiffness of kT, $h$ is the height of the elastic gasket and has the following calculation formula,

4 $$k_{T} = E_{T} A_{T} /h$$

At this time, the additional opening of the joint of the shield segment assembly structure caused by the water pressure is Δx1. Because the upper and lower sides of the joint of the shield segment assembly structure will have different openings due to external factors, the total openings of the upper and lower sides of the structural joint are Δx1 and Δx2, respectively.

5 $$\Delta T_{1} = k_{T} \Delta {\text{x}}_{{1}}$$

6 $$\Delta T_{2} = k_{T} \Delta {\text{x}}_{{2}}$$

**Figure 2:**
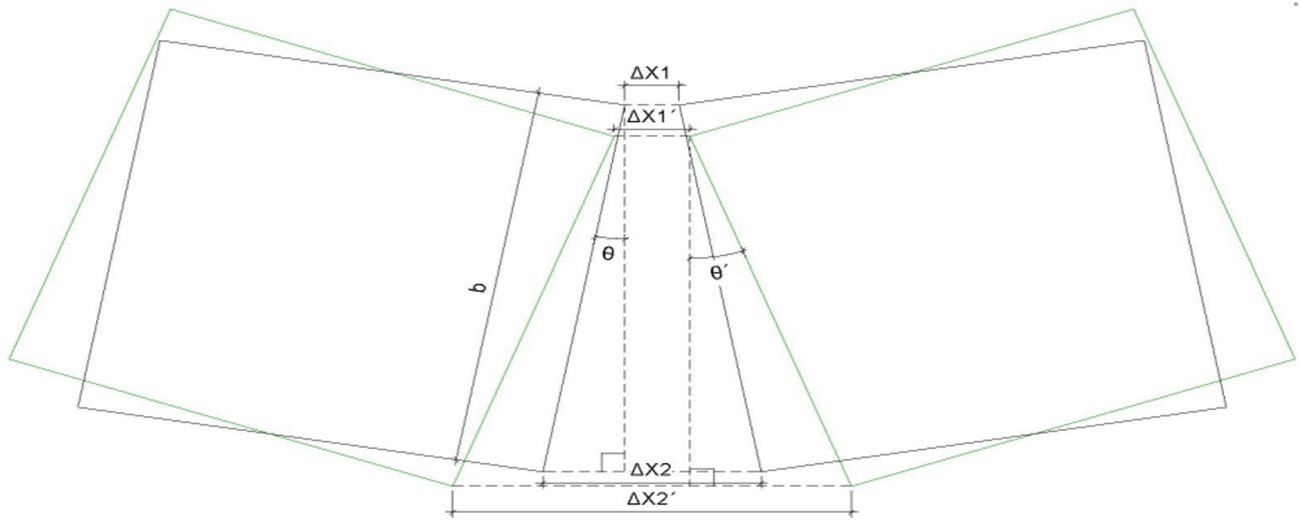
Schematic diagram of upper and lower openings of segment structure joints.

If ∆ × 1 is the opening of the upper joint, ∆ × 2 is the amount of opening on the lower side of the joint of the corresponding structure that is different. That is, the corresponding structure deviates to the inside, as shown in Fig. 2, in which ∆ × 1 and ∆ × 2. For the opening of the structural joint at θ angle, ∆ × 1’ and ∆ × 2´ is the opening amount of the structural joint when the structure is offset to θ´, and b is the height of the structural joint, which is obtained by the geometric relationship

7 $$2b\sin \theta + \Delta {\text{x}}_{1} = \Delta {\text{x}}_{2}$$

Equations (5), (6), (7), and substitution Formula (3) are available.

8 $$\Delta {\text{x}}_{{1}} = \frac{{P_{w} A_{w} - 2k_{T} {\text{bsin}}\theta }}{{2k_{T} }}$$

9 $$\Delta {\text{x}}_{{2}} = \frac{{P_{w} A_{w} + {2}k_{T} {\text{bsin}}\theta }}{{{2}k_{T} }}$$

Equations (8), (9) into (5) and (6) are available.

10 $$\Delta {\text{T}}_{{1}} = k_{T} \frac{{P_{w} A_{w} - {2}k_{T} {\text{bsin}}\theta }}{{{2}k_{T} }}$$

11 $$\Delta {\text{T}}_{{2}} = k_{T} \frac{{P_{w} A_{w} + {2}k_{T} {\text{bsin}}\theta }}{{{2}k_{T} }}$$

**Figure 3:**
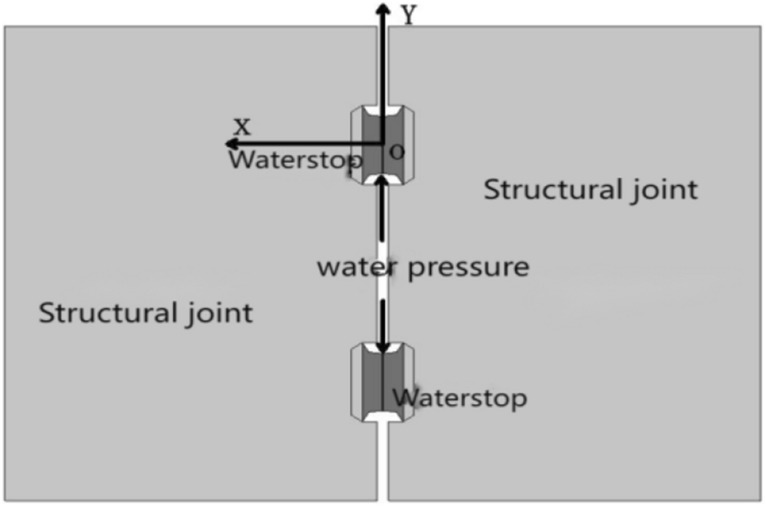
Stress diagram of sealing waterstop under water pressure.

The unit body of the sealing waterstop is selected for analysis, as shown in Fig. 3.

When the joint is not affected by water pressure, from the initial conditions and boundary conditions,

12 $$\left\{ {\begin{array}{*{20}l} {\sigma _{{x{1}}} = \sigma _{{{\text{01}}}} } \hfill \\ {\sigma _{{x{2}}} = \sigma _{{{\text{0}}{2}}} } \hfill \\ {\sigma _{y} = 0} \hfill \\ {\sigma _{z} = \sigma _{0} } \hfill \\ {\varepsilon _{{x1}} = \varepsilon _{{{\text{01}}}} = \Delta h_{1} /h} \hfill \\ {\varepsilon _{{x{2}}} = \varepsilon _{{{\text{0}}{2}}} = \Delta h_{{2}} /h} \hfill \\ \end{array} } \right.$$

Substituting Eq. (12) into the physical equation of elastic mechanics of the element body^21^, we can obtain

13 $$E_{T} \varepsilon_{01} = \sigma_{01} - \upsilon \sigma_{0} = \sigma_{01} \left( {1 - \upsilon^{2} } \right)$$

14 $$E_{T} \varepsilon_{02} = \sigma_{02} - \upsilon \sigma_{0} = \sigma_{02} \left( {1 - \upsilon^{2} } \right)$$

When the shield segment assembly joint is subjected to water pressure with a pressure of pw, considering the friction between the upper and lower surfaces, there are

15 $$\left\{ {\begin{array}{*{20}l} {\sigma _{{x{1}}} = \sigma _{{p{\text{1}}}} } \hfill \\ {\sigma _{{x{2}}} = \sigma _{{p{2}}} } \hfill \\ {\sigma _{y} = p_{{\text{w}}} } \hfill \\ {\varepsilon _{{x{1}}} = \varepsilon _{{{\text{01}}}} = \left( {\varepsilon _{{01}} h_{{1}} - \Delta x_{1} } \right)/h_{1} } \hfill \\ {\varepsilon _{{x{2}}} = \varepsilon _{{{\text{0}}{2}}} = \left( {\varepsilon _{{0{2}}} h_{{2}} - \Delta x_{{2}} } \right)/h_{{2}} } \hfill \\ {\sigma _{z} = \sigma _{f} } \hfill \\ \end{array} } \right.$$

Substituting Eq. (15) into the physical equation of elastic mechanics, we can obtain that

16 $$E_{T} \varepsilon_{01} - E_{T} \frac{{\Delta x_{1} }}{{h_{{}} }} = \sigma_{p1} \left( {1 - \upsilon^{2} } \right) - p_{w} \left( {1 + \upsilon } \right)\upsilon$$

17 $$E_{T} \varepsilon_{02} - E_{T} \frac{{\Delta x_{2} }}{h} = \sigma_{p2} \left( {1 - \upsilon^{2} } \right) - p_{w} \left( {1 + \upsilon } \right)\upsilon$$

Simultaneous Eqs. (8), (9), (13), (14), (16), and (17), we obtain

18 $$\sigma_{p1} = \sigma_{01} + p_{w} \left[ {\frac{\upsilon }{{\left( {1 - \upsilon } \right)}} - \frac{{E_{T} A_{w} }}{{2k_{T} h\left( {1 - \upsilon^{2} } \right)}}} \right] + \frac{{E_{T} b\sin \theta }}{{h\left( {1 - \upsilon^{2} } \right)}}$$

19 $$\sigma_{p2} = \sigma_{02} + p_{w} \left[ {\frac{\upsilon }{{\left( {1 - \upsilon } \right)}} - \frac{{E_{T} A_{w} }}{{2k_{T} h\left( {1 - \upsilon^{2} } \right)}}} \right] - \frac{{E_{T} b\sin \theta }}{{h\left( {1 - \upsilon^{2} } \right)}}$$

Substituting Eqs. (4) into (18) and (19), the contact compressive stress on the surface of the sealing waterstop is obtained as follows

20 $$\sigma_{p1} = \sigma_{01} + p_{w} \left[ {\frac{\upsilon }{{\left( {1 - \upsilon } \right)}} - \frac{{A_{w} }}{{2\left( {1 - \upsilon^{2} } \right)A_{T} }}} \right] + \frac{{k_{T} b\sin \theta }}{{\left( {1 - \upsilon^{2} } \right)A_{T} }}$$

21 $$\sigma_{p2} = \sigma_{02} + p_{w} \left[ {\frac{\upsilon }{{\left( {1 - \upsilon } \right)}} - \frac{{A_{w} }}{{2\left( {1 - \upsilon^{2} } \right)A_{T} }}} \right] - \frac{{k_{T} b\sin \theta }}{{\left( {1 - \upsilon^{2} } \right)A_{T} }}$$

Perhaps

22 $$\sigma _{{p1}} = \sigma _{{01}} + p_{w} \frac{\upsilon }{{\left( {1 - \upsilon } \right)}} + \frac{1}{{2\left( {1 - \upsilon ^{2} } \right)A_{T} }}\left( {2k_{T} b\sin \theta - p_{w} A_{w} } \right)$$

23 $$\sigma_{p2} = \sigma_{02} + p_{w} \frac{\upsilon }{{\left( {1 - \upsilon } \right)}} - \frac{1}{{2\left( {1 - \upsilon^{2} } \right)A_{T} }}\left( {2k_{T} b\sin \theta + p_{w} A_{w} } \right)$$

In the formula,

$\sigma_{01}$—the compressive stress on the surface of the upper side gasket before the action of water pressure.

$\sigma_{02}$—the compressive stress on the surface of the lower segment gasket before the action of water pressure.

$p_{w}$—the size of the water pressure.

$A_{w}$—the water pressure area along the axial direction of the joint.

$A_{T}$—the contact area of the sealing waterstop $k_{T}$.

$\upsilon$—compression line stiffness and Poisson’s ratio of the sealing waterstop.

$b$—structural joint height.

$\theta$—structural offset angle.

It can be seen from the contact compressive stress formula that the waterproof performance of the gasket depends not only on its own contact area, linear compression stiffness, and Poisson’s ratio but also on the size of the joint opening caused by the height of the segment joint specimen and the inclination angle. As the Poisson’s ratio of the gasket increases, the waterproof performance of the gasket improves. When the height of the structure is constant, increasing the inclination angle of the structural joint will increase the extrusion sealing resistance of the upper gasket and improve the waterproof performance. An increase in the water pressure of the upper-side gasket will result in a decrease in the extrusion sealing resistance of the lower-side gasket. Therefore, the inclination angle of the structural joint has a significant impact on the waterproof performance of the gasket. However, a larger inclination angle of the structural joint will result in a larger assembly space, which can easily damage the edge angles and end faces of the segment joint. Therefore, the inclination angle should be adjusted with an appropriate offset rate.

According to the sealing principle, the contact compressive stress on the surface of the gasket is an important index to measure its waterproof performance^20^. For a given gasket, the greater the contact compressive stress on the surface of the gasket, the higher the water pressure on the gasket’s surface.

## Numerical simulation

Based on the ABAQUS finite element software, this study examines the mechanical properties of tunnel segment joint gaskets under different forms of segment dislocation and opening. It also analyzes the influence of relevant parameter indexes of segment joint gaskets on their mechanical state, providing a basis for studying the waterproof performance of tunnel segment joint gaskets.

### Model building

**Figure 4:**
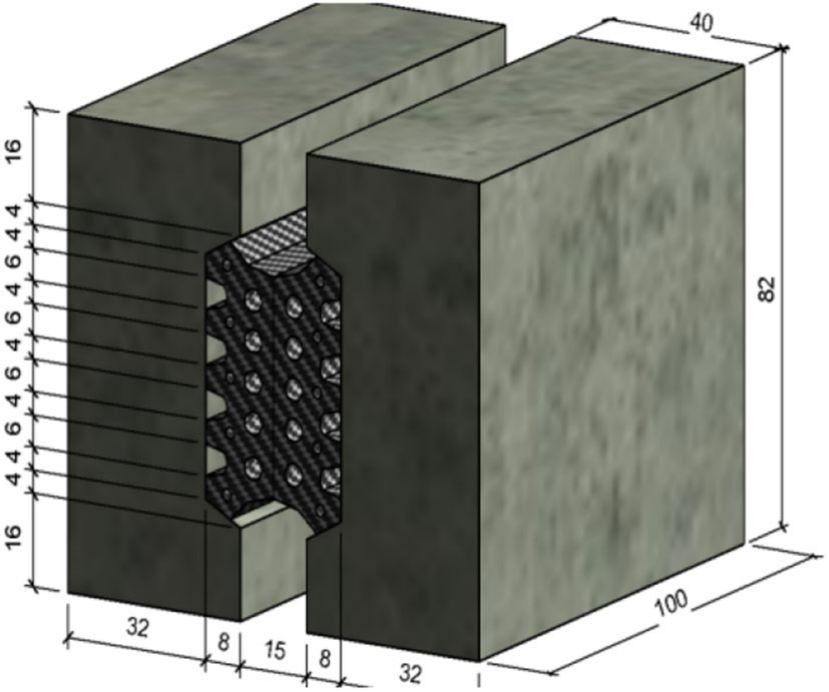
Schematic diagram of three-dimensional model of segment waterstop.

The model calculates and controls a single variable, considers the settlement and deformation of the shield tunnel segment structure under different load conditions and the joint sealing materials at different positions, that is, different segment openings and dislocations, different water pressures, and analyzes the mechanical effects of related variables on the waterproofing of segment joints. According to different control variables, this section establishes the contact stress of the longitudinal sealing gasket of the segment under different load forces. The model size is shown in Fig. 4, and the units marked in the figure are mm.

In the model, C50 concrete is used for the segment lining with an elastic modulus of 34.5 GPa and a Poisson’s ratio of 0.167. The concrete is an isotropic elastic material, and the segment material is modeled as damaged plastic. The EPDM rubber gasket material is selected for the segment joint. The rubber gasket material follows the Mooney–Rivlin model and is considered a compressible isotropic hyperelastic material^22^. The elastic and hyperelastic materials are modeled using the C3D8RH (C represents solid element, 3D represents 3D element, 8 represents 8 nodes) eight-node linear hexahedron element, while the cohesive element is modeled using the COH3D8 (COH stands for adhesive unit) eight-node three-dimensional bonding element.

**Figure 5:**
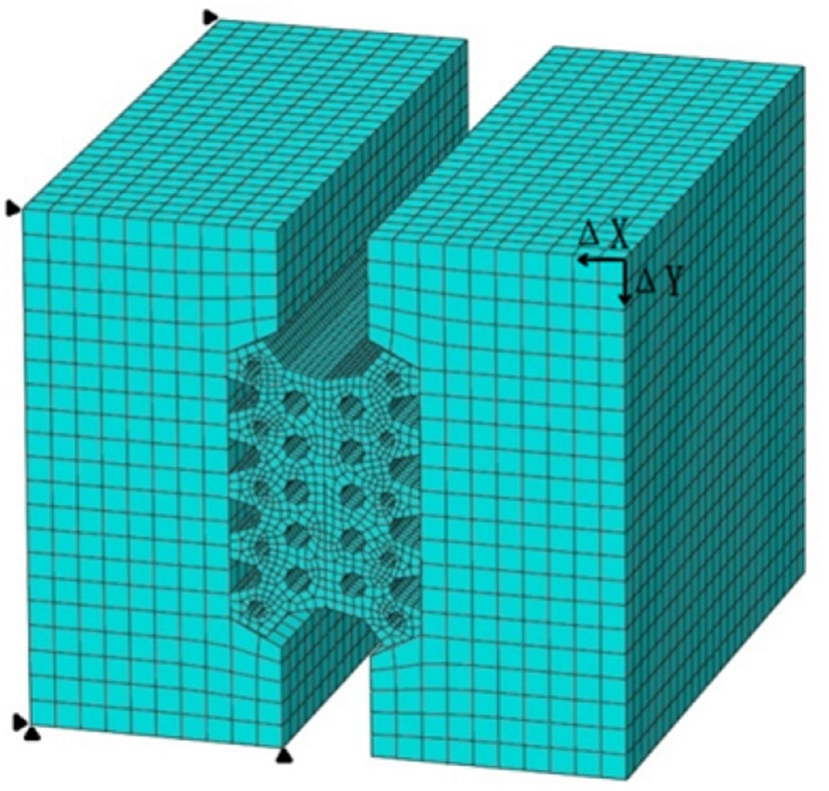
Model load and boundary diagram of waterstop.

The left concrete body of the model is completely fixed as a boundary condition. Dislocations ΔX and ΔY in the X and Y directions are applied to the right concrete body to simulate the opening and dislocation of the segment. The hydrostatic pressure is applied to the upper boundary of the hyperelastic element and the cohesive element through pressure P (as shown in Fig. 5).

### Analysis of different longitudinal dislocation results of segment joints

For the calculation and comparison of different segment dislocations, the segment joint gasket model selects the EPDM rubber waterstop belt model, changes the size of the segment dislocation and opening, and the hydrostatic pressure is 0.1 MPa unchanged. The stress state of the contact surface of the segment sealing belt is analyzed. Generally, the compressive stress of the segment is positive in the tunnel project, so the compressive stress is positive here.

**Figure 6:**
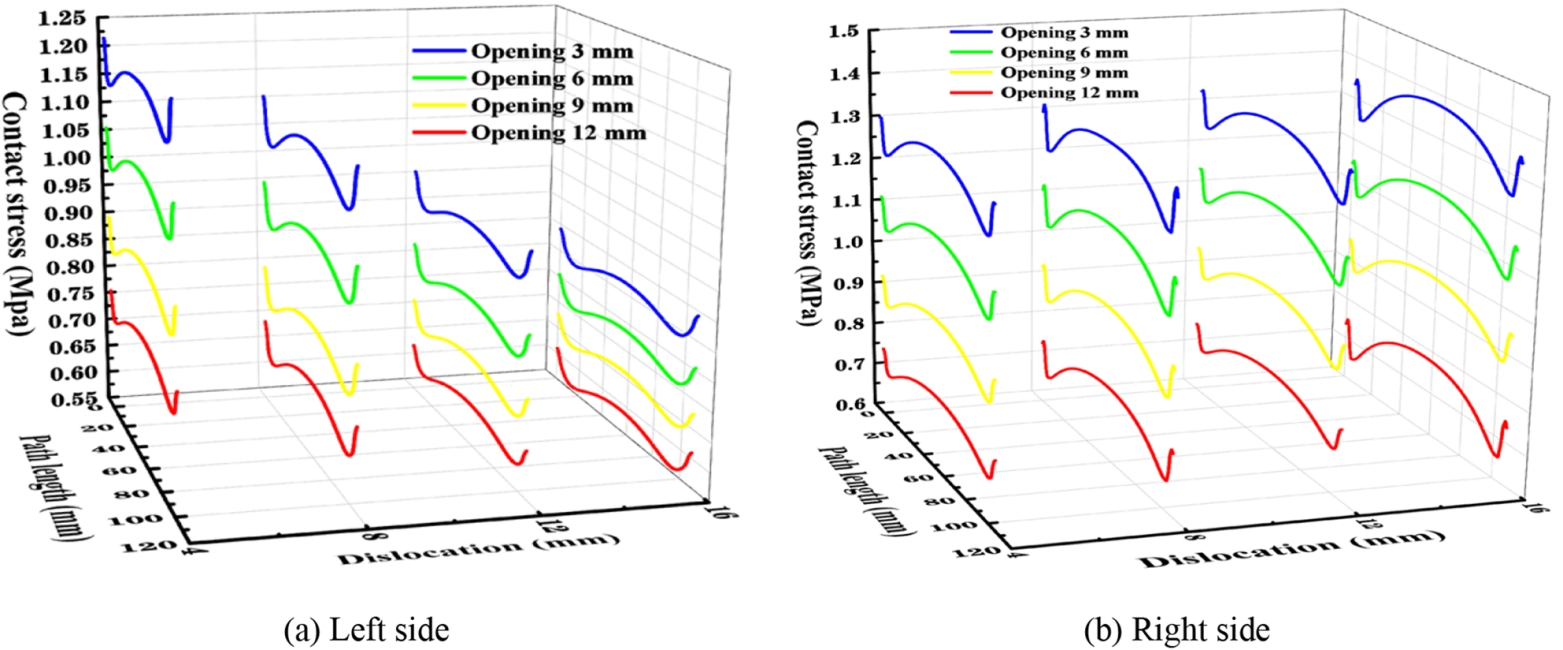
The stress under different openings and dislocations of the longitudinal left and right joints of the segment gasket.

From Fig. 6 combined with Table 1, it can be seen that the longitudinal stress at the left and right joints of the segment gasket decreases as the opening amount increases. In Fig.a, the longitudinal contact stress of the 12 mm and 16 mm staggered segment gaskets decreased slightly less than that of the 4 mm and 8 mm dislocations, with an average decrease of 0.144 MPa and 0.114 MPa at 4 mm and 8 mm, and an average decrease of 0.105 MPa and 0.077 MPa at 12 mm and 16 mm.According to figure b, the average increase of contact stress under different dislocations is 0.144 MPa for 4 mm, 0.187 MPa for 8 mm, 0.193 MPa for 12 mm and 0.199 MPa for 16 mm. The decrease of longitudinal contact stress of segment gasket increases with the increase of dislocation. At the dislocation of 16 mm, the contact stress on the right side is about 50% higher than that on the left side. This shows that with the decrease of contact area between gaskets, the change of opening amount has little influence on the longitudinal left contact stress of segment gasket, but has great influence on the longitudinal right contact stress. On the other hand, in Fig.a, the stress of the left longitudinal joint of the segment gasket decreases with the increase of the dislocation. The dislocation stress at 16 mm is 35% lower than that at 4 mm, and the decrease of the longitudinal contact stress amplitude decreases with the increase of the dislocation. In figure b, the stress at the longitudinal right joint of the segmental gasket increases with the increase of the dislocation. The dislocation stress at 16 mm is 2% higher than that at 4 mm, and the longitudinal contact stress increases with the increase of the dislocation. The amount of increase decreases with the increase of the opening amount.

**Table 1:** The mean stress of different openings and dislocations on both sides of the longitudinal joints MPa.

Quantity	4 mm		8 mm		12 mm		16 mm	
	Left	Right	Left	Right	Left	Right	Left	Right
3 mm	1.182	1.294	1.066	1.298	0.925	1.322	0.807	1.334
6 mm	1.033	1.109	0.915	1.112	0.794	1.134	0.724	1.138
9 mm	0.879	0.922	0.794	0.925	0.692	0.939	0.650	0.942
12 mm	0.750	0.746	0.724	0.738	0.609	0.742	0.575	0.736
Average	0.961	1.018	0.875	1.019	0.755	1.034	0.689	1.038

**Figure 7:**
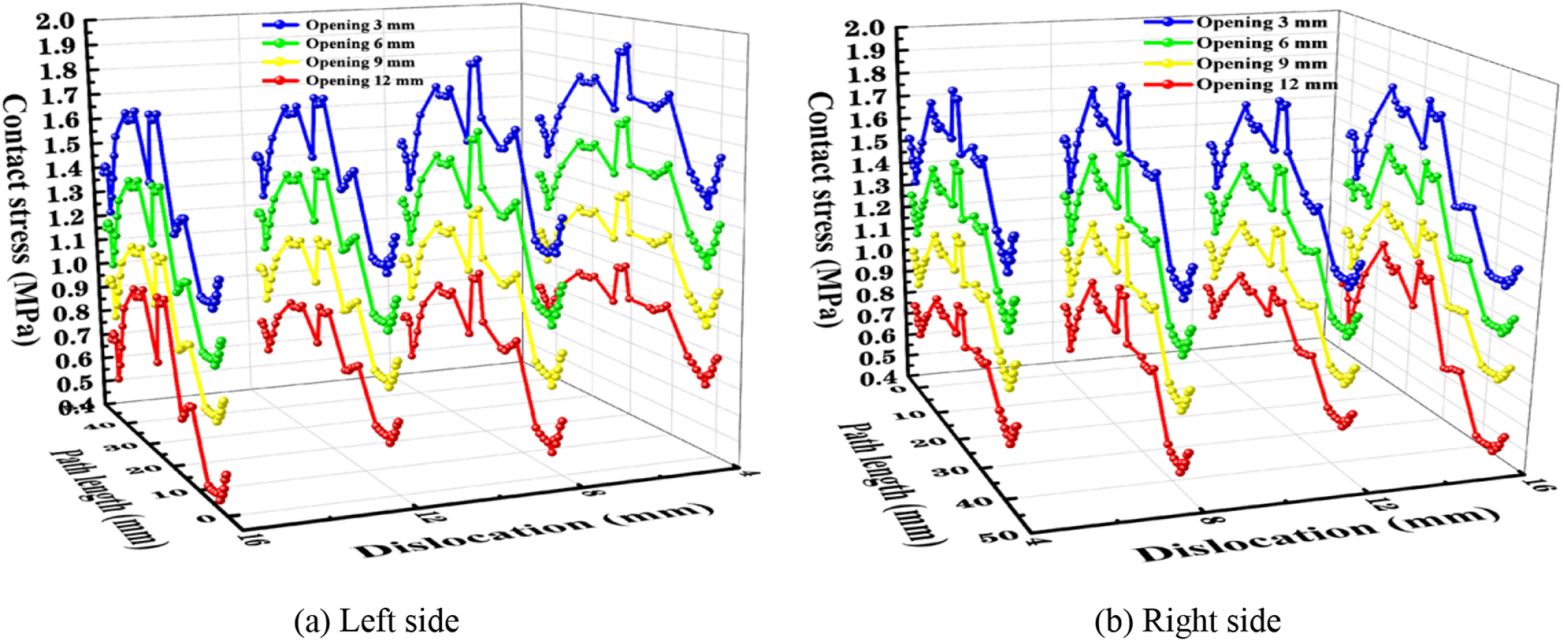
The contact stress of the left and right joints of the segment gasket section under different openings and dislocations.

From Fig. 7 and Table 2, it can be seen that the stress at the dislocation of 8 mm is 0.094 MPa lower than that at the dislocation of 4 mm, and the stress at the dislocation of 16 mm is 0.035 MPa lower than that at the dislocation of 12 mm, indicating that the stress reduction of the cross-section contact stress decreases with the increase of the amount of dislocation. For the same stress curve, the stress at the beginning of the stress path in the left joint is less than that at the end of the stress path, and the stress at the beginning of the stress path in the right joint is greater than that at the end of the stress path. It can be seen that the squeezing force of the sealing gasket at the top of the left joint is less than that at the top of the right joint. When the contact area of the sealing gasket section is large, the change of the opening amount has an effect on the contact stress on the left and right sides of the sealing gasket section. When the squeezing force of the sealing gasket is large, the dislocation change has an effect on the contact stress on the left and right sides of the sealing gasket section. Through the overall analysis, it can be seen that the stress curve changes little with the change of dislocation, while the stress curve changes greatly with the change of opening amount. The mean value of stress on the left side at 12 mm is 47.2% lower than that at 3 mm, and the right side is 49.6% lower, indicating that the influence of opening amount on the contact stress change of segment gasket is greater than that of dislocation.

**Table 2:** The mean value of stress curve under different openings and dislocations MPa.

Quantity	4 mm		8 mm		12 mm		16 mm		Average	
	Left	Right	Left	Right	Left	Right	Left	Right	Left	Right
3 mm	1.578	1.582	1.484	1.506	1.451	1.433	1.407	1.415	1.480	1.484
6 mm	1.325	1.328	1.228	1.247	1.214	1.204	1.177	1.188	1.236	1.242
9 mm	1.067	1.070	0.973	0.983	0.980	0.974	0.948	0.953	0.992	0.995
12 mm	0.811	0.814	0.717	0.721	0.743	0.762	0.919	0.693	0.748	0.748
Average	1.195	1.199	1.101	1.114	1.097	1.093	1.062	1.062	1.114	1.117

## Model experiments

In order to meet the waterproof design requirements of tunnel underground engineering and ensure the accuracy of test data, this paper uses a steel device to carry out a waterproof test on the seaming material and sets the segment structure joint waterproof for the joint gasket waterproof.

### The overall scheme of the experimental system

**Figure 8:**
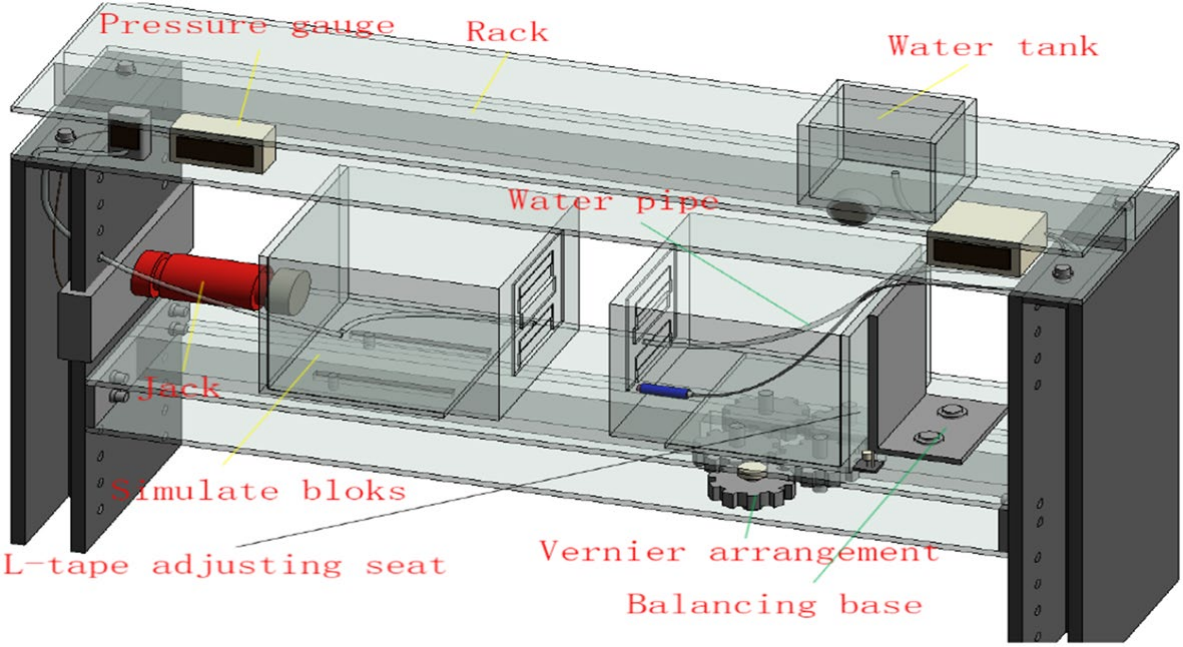
The schematic diagram of the overall experimental scheme of the 1 : 1 thickness of the segment.

The overall scheme of the test system for the joint size of the tunnel segment structure designed according to the thickness of the segment of a subway tunnel in Shenzhen is shown in Fig. 8. The test system includes a model test piece, water injection device, stabilization device, and monitoring system^23^.

The model test piece includes two segment joint test blocks, a detachable rubber waterstop, a water tank, a water pressure meter, and a transparent rubber water pipe. The water pressure strength under different water pressures is provided for the tunnel segment joint test block. The stabilizing device includes a jack, an I-beam, and a jack counterforce frame. It is used for surface water seepage detection at different joint distances of tunnel segments and water expansion detection of the detachable rubber waterstop.

The monitoring system consists of a force sensor, a humidity sensor, a pressure gauge, a barometer, a water pressure gauge, and an intelligent terminal humidity display. The force sensor is connected to the pressure gauge. The barometer is displayed inside the wireless numerical control inflatable pump. The intelligent terminal humidity display monitoring equipment is considered to be installed in the I-beam at the top of the overall frame in the same way as the pressure gauge, the barometer, and the water pressure gauge. The specific placement position of the monitoring digital display device can also be replaced according to the on-site implementation. This test is mainly for the surface seepage detection of the tunnel segment joint joints under different dislocations and openings. It is also for the water absorption detection of the structural joint specimen material and the water expansion detection of the detachable rubber waterstop.

### Program implementation process

**Figure 9:**
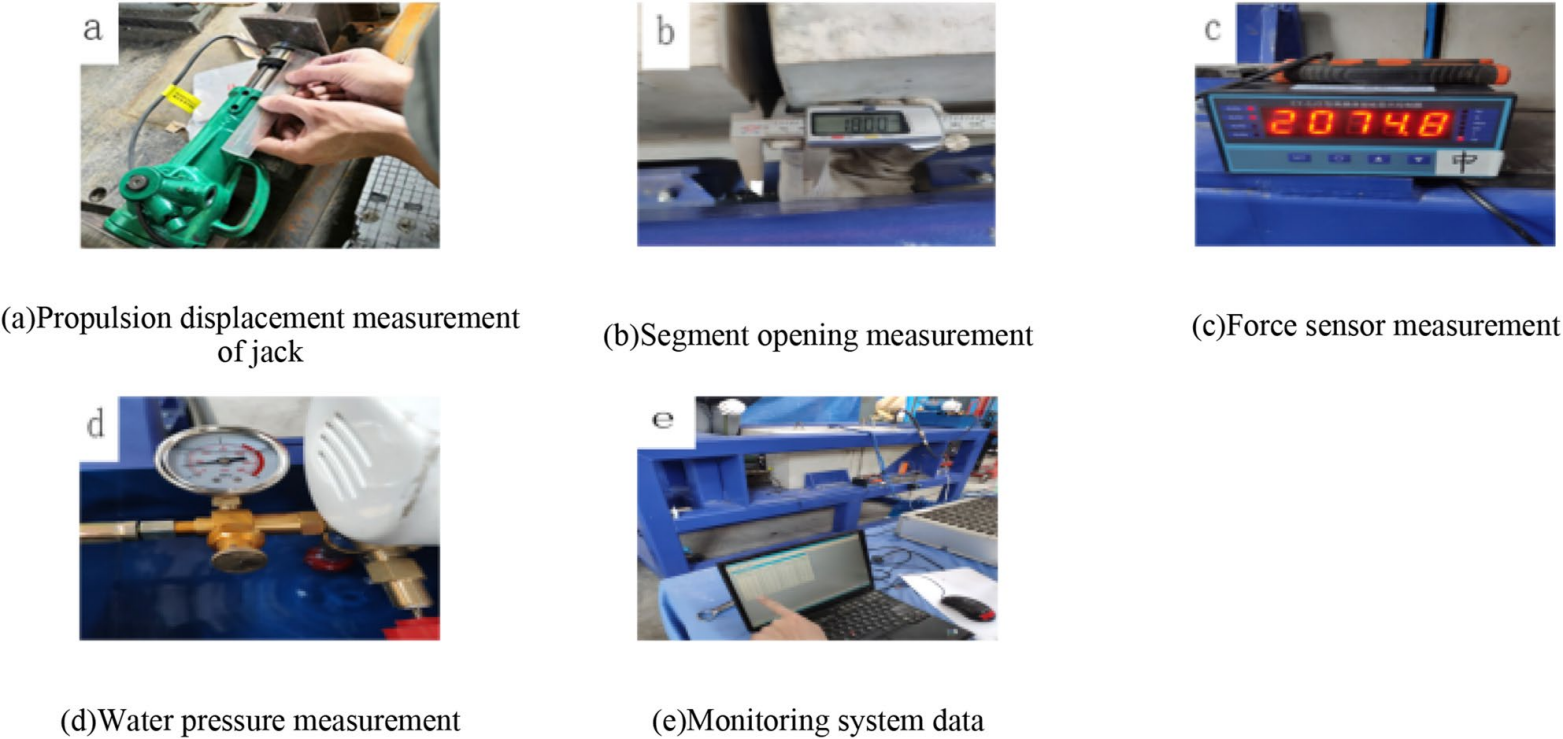
Experimental monitoring and implementation process.

The test implementation process is shown in Fig. 9. According to the preset distance of the joint after the splicing of the tunnel segment joint specimen, the maximum opening amount of the segment joint is required to be 12 mm. The left test block is pushed to the right by jack pressurization, and the waterstops are in contact with each other. The axial pressure causes the right test piece to press against the side wall of the experimental platform, resulting in the squeezing force of the waterstop and achieving the preset distance of 12 mm, 11 mm, and 2 mm, as shown in Fig. 9a. At the same time, through the vertical braking micro-motion device on the right side of the experimental platform, four bolts are rotated to make the specimen rise and fall while the precise dislocation distance is increased from 3 to 15 mm in turn. As shown in Fig. 9b, the preset joint distance is controlled in turn by the digital vernier caliper. The left side of the left specimen is connected to the force sensor and the steel plate. The left side of the force sensor realizes the dynamic balance of the force sensor, the steel plate and the test block through the jack. The force sensor is connected to the pressure gauge through the transmission line, as shown in Fig. 9c the dynamic balance of the pressure gauge monitoring device. In the right test block, the water injection pipe is connected to the outlet pipe of the water tank, and the connection position of the water pressure gauge is between the water tank and the outlet pipe. The water injection pipe of the water injection device is equipped with a water pressure gauge to detect the water pressure during water injection. As shown in Fig. 9d, the initial water pressure is preset according to different openings and dislocations. Each stage is pressurized by 0.05 MPa. When the water pressure gauge reaches the design water pressure, the system stabilizes the water pressure for 12 min, and the pressure is pressurized until the sealing gasket waterproof performance fails under each opening and dislocation. At the same time, the static strain measurement system is used to monitor the balance sensor of the equipment, as shown in Fig. 9e.

**Figure 10:**
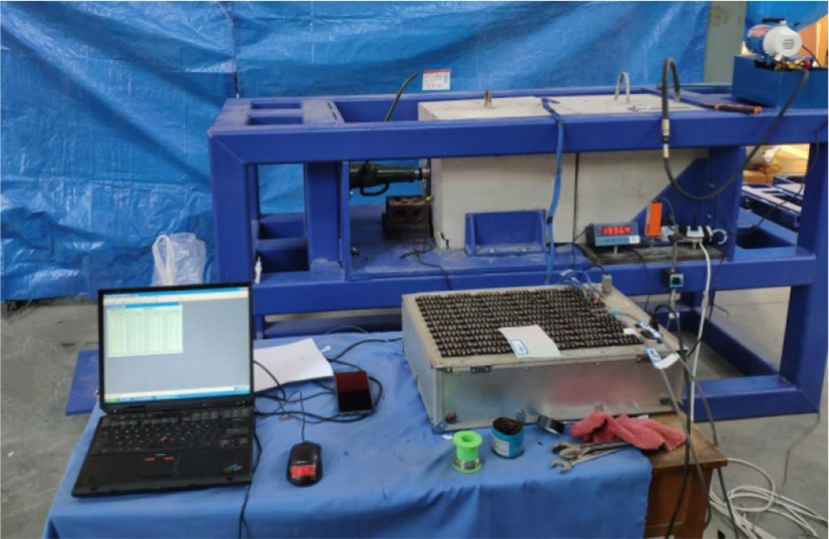
The overall operation implementation diagram of the experiment.

The overall operation of the test is shown in Fig. 10. Five people are required to control the test operation process at the same time. Two people lift the right test block vertically through the rotating bolt of the vertical braking micro-motion device. One person operates the jack to apply the axial pressure. One person measures the joint opening of the test block and observes the reading of the pressure gauge at the same time. One person monitors the balance sensor indication of the equipment through the static strain measurement system.

### Analysis of the data results

**Figure 11:**
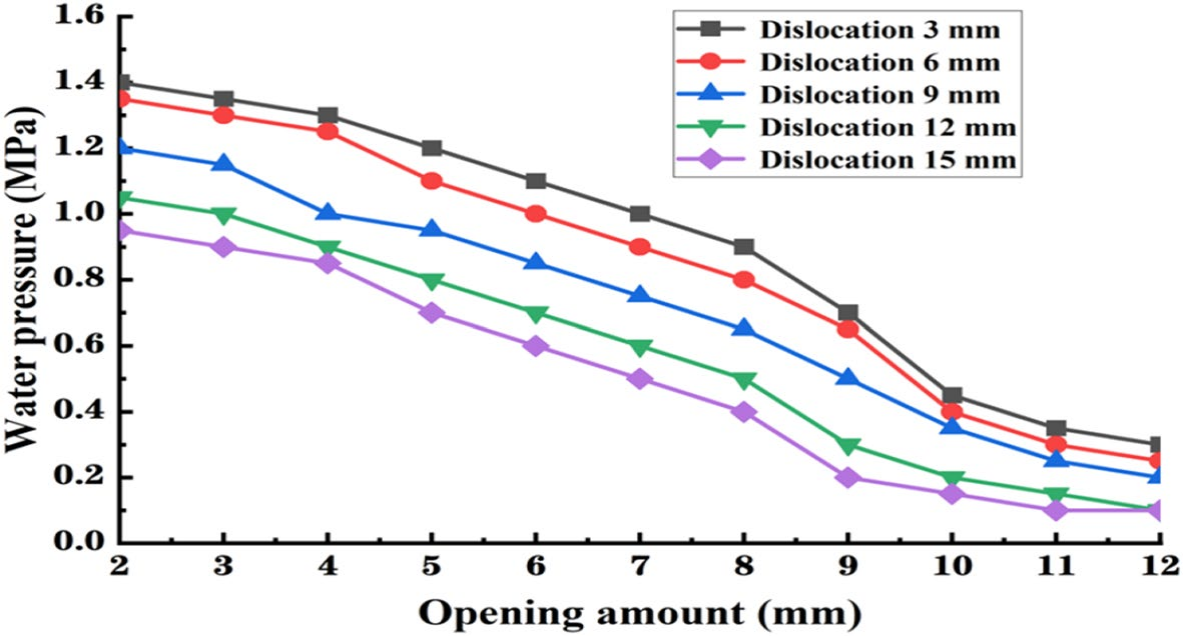
The relationship curve between different joint dislocations and water pressure resistance.

It can be seen from Fig. 11 that the water resistance pressure gradually decreases with the increase of the opening amount of the segment gasket. The curves of water resistance pressure show a linear downward trend with different slopes for the opening ranges of 2–4 mm and 4–8 mm. After the segment joint is increased to 8 mm opening, the decrease of water resistance pressure is obviously increased, and the descending curvature in the opening range of 8–10 mm is significantly larger than that in the previous range. When the opening amount is 10–12 mm, the water resistance pressure tends to stabilize and gradually become stable. In terms of segment dislocation, the water resistance pressure gradually decreases with the increase of the dislocation amount. The curves for dislocations of 3 mm and 6 mm are consistent. However, for a dislocation of 9 mm, the water pressure curve decreases significantly compared to the previous two, and there is a large pressure difference between the upper and lower parts. The water resistance pressure curves for dislocations of 12 mm and 15 mm are similar, with a small pressure difference. The overall drop rate of water pressure is greater for opening than for dislocation, indicating that the opening has a greater influence on the waterproof performance of the tunnel segment joint gasket than the dislocation.

### Simulation test comparison

**Figure 12:**
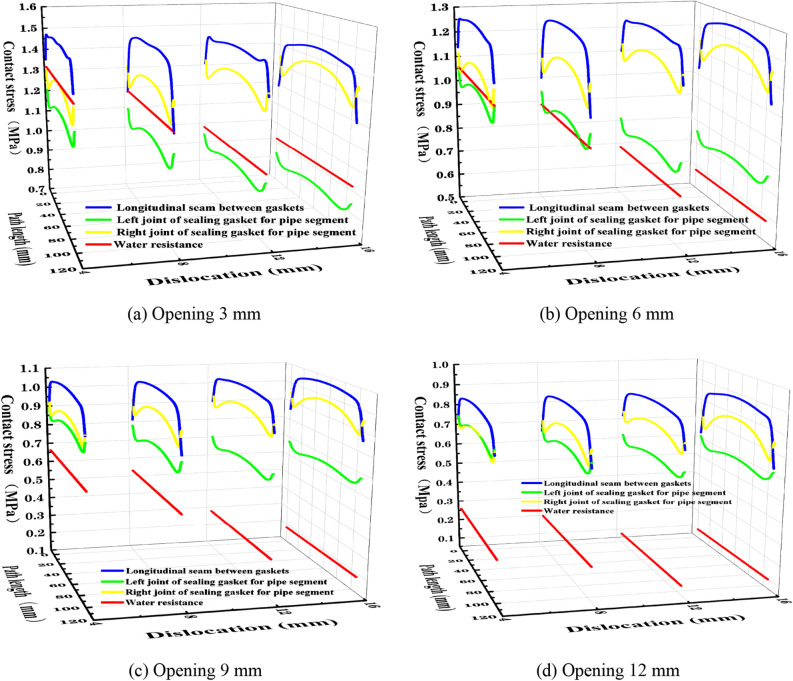
Relationship between longitudinal joint stress and water resistance pressure of gasket.

Through the numerical simulation in the third section, the contact stress distribution of different joints of the segment gasket is obtained, and the results are compared with the water resistance pressure of the segment gasket under different joints in the prototype waterproof test results, as shown in Fig. 12.

According to Fig. 12, when the opening of the segment joint is 3 mm, the water resistance pressure under the dislocation of 4 mm is greater than the contact stress of the left and right sides of the segment gasket but less than the contact stress of the indirect seam of the gasket. For the dislocation of 8 mm, the water resistance pressure is greater than the contact stress of the left seam of the segment gasket and has an intersection with the contact stress of the indirect seam. When the dislocation is 12 mm or 16 mm, the water resistance pressure is only greater than the contact stress of the left seam of the segment gasket and less than the contact stress between the gasket and the right side. For the opening amount of 6 mm, the water resistance pressure under the dislocation of 4 mm is greater than the contact stress of the left joint of the segment gasket and has an intersection with the contact stress of the right joint. Under the dislocation of 8 mm, the water resistance pressure has an intersection with the contact stress of the left joint and is less than the contact stress between the gasket and the right joint. The water resistance pressure for other opening amounts and dislocations is less than the contact stress between the longitudinal left and right sides of the segment gasket and the gasket.

In order to compare and quantify the contact stress of the segment gasket joint with the water resistance pressure, the passing rate of the waterproof performance of the segment gasket under the condition that the contact stress of the segment gasket joint is less than the water resistance pressure position is listed, as shown in Table 3.

**Table 3:** Pass rate of contact stress waterproof performance of segment gasket joints.

Waterproof parate (%)	Segment sealing	Pipe seal pad	Pipe seal pad
	Gasket room	Longitudinal left joint	Longitudinal right joint
Open 3 dislocation 4	100	0	0
Open 3 dislocation 8	98.01	0	100
Open 3 dislocation 12	100	0	100
Open 3 dislocation 16	100	0	100
Open 6 dislocation 4	100	0	100

**Figure 13:**
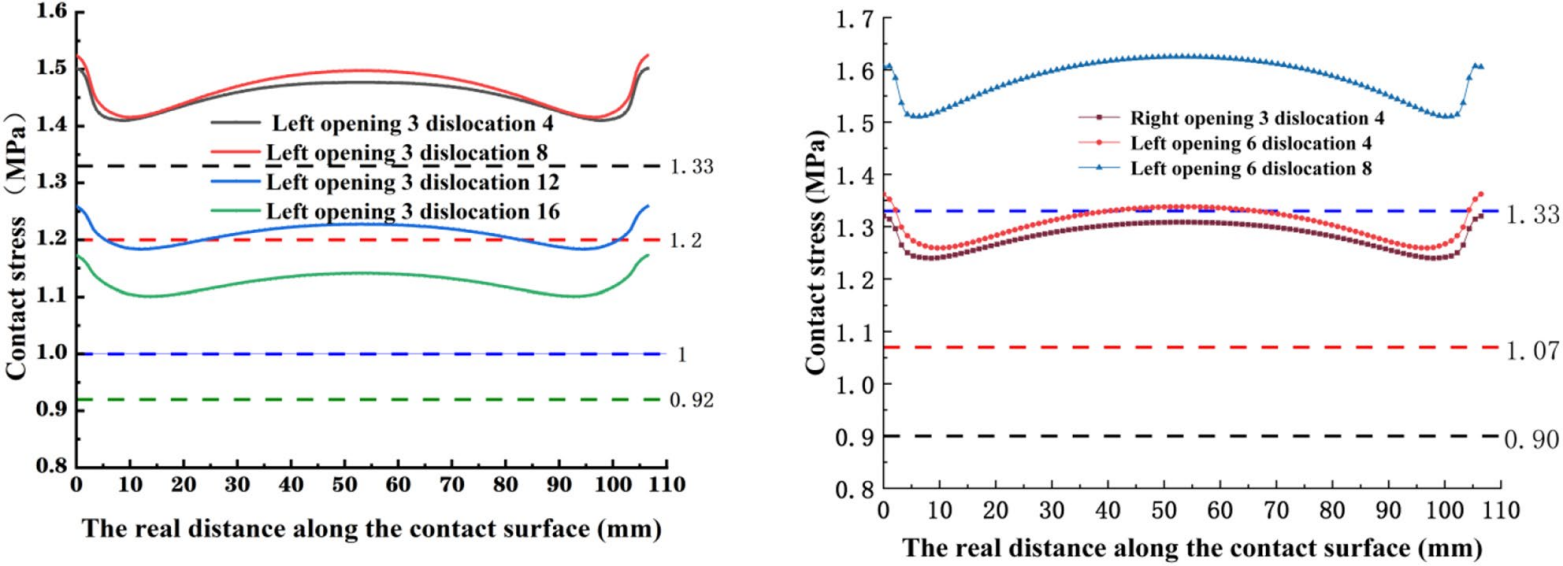
Corrected stress and water resistance pressure of longitudinal joint of gasket.

Through the above analysis, it can be seen that the longitudinal left joint of the segment gasket is the weakest joint in waterproof performance. There is water pressure passing through the upper joint under different dislocations of 3 mm and 6 mm and dislocations of 4 mm and 8 mm, but it can not be explained that the water flow enters the inside of the tunnel through the left joint under these dislocations. The contact stress value of the left joint of the segment gasket section is different. In theory, as long as the maximum contact stress value of the left joint of the section is greater than the water resistance pressure, the waterproof effect can be achieved. By taking the maximum contact stress value of the segment gasket section as the amplification factor of the longitudinal contact stress curve of the segment gasket, the starting point value of the stress curve of the left joint of the segment gasket section is defined as the Lvalue. The longitudinal contact stress of the segment gasket is corrected, as shown in Table 4 and Fig. 13.

**Table 4:** Contact stress coefficient for tube fitting sealing cushion joint correction condition.

Correcting working conditions	Least value (MPa)	Crest value (MPa)	Amplification coefficient
Open 3 dislocation 4	1.53828	1.88981	1.228521466
Open 3 dislocation 8	1.35921	1.86612	1.372944578
Open 3 dislocation 12	1.33824	1.7514	1.308733859
Open 3 dislocation 16	1.23921	1.72716	1.393758927
Open 6 dislocation 4	1.28878	1.5989	1.240630674
Open 6 dislocation 8	1.11148	1.58686	1.427700004
Open 3 dislocation 4 (vertical right)	1.54995	1.89517	1.222729765

The corresponding color dotted line represents the water resistance pressure corresponding to the contact stress curve. It can be observed from Fig. 13 that the contact stress of the longitudinal left and right sides of the modified segment gasket is greater than the corresponding water resistance pressure value. At this time, the waterproof performance pass rate of the segment gasket’s contact stress is 100%. However, considering the contingency of the maximum contact stress, if the waterproof performance pass rate is set as 100%, the contact stress and water resistance pressure on the entire joint of the cross-section are quantitatively compared, and the waterproof rate of the gasket joint is analyzed. The pass rate of 100% means that the minimum value on the longitudinal contact stress curve of the gasket is greater than or equal to the water resistance pressure. The absolute value of the difference between the two is added to the left value of the contact stress of the gasket section, and this value is defined as the A value. The A value is compared with the contact stress value of the gasket section. The value greater than or equal to the A value on the stress curve is the waterproof pass rate value, as shown in Table 5. The minimum value in the represents the minimum value on the longitudinal contact stress curve of the gasket.

**Table 5:** Waterproof rate of segment gasket joint under modified working conditions.

Correcting working conditions	Least value (MPa)	Water resistance pressure (MPa)	Differentials (MPa)	A value (MPa)	Waterproof rate (%)
Open 3 dislocation 4	1.14763	1.33	0.18237	1.72065	40
Open 3 dislocation 8	1.031	1.2	0.169	1.52821	45.71
Open 3 dislocation 12	0.90446	1	0.9554	1.43378	51.43
Open 3 dislocation 16	0.79002	0.92	0.12998	1.36919	57.14
Open 6 dislocation 4	0.99913	1.07	0.07087	1.35965	42.86
Open 6 dislocation 8	0.88184	0.9	0.01816	1.12964	65.71
Open 3 dislocation 4 (vertical right)	1.23533	1.33	0.09467	1.64462	42.86

From Table 3, it is known that the waterproofing rate of the joint section of the segment gasket section is greater than 40% under the modified working condition. From Fig. 7, it is known that the larger contact stress of the joint section of the segment gasket section is distributed in the middle of the curve, and the water flow is blocked from top to bottom through the segment gasket section up to 30% of the joint path. It can be seen that the waterproof performance of the joint section of the segment gasket is good, and the contact stress of the segment gasket is greater than the water resistance pressure. In summary, the numerical simulation results are basically consistent with those obtained in the waterproofing experiment.

## Conclusions

In this paper, a mechanical calculation model is first constructed for theoretical analysis, and then the longitudinal section of the segment joint gasket model under different opening dislocations is analyzed by numerical simulation. Finally, the waterproof performance of the tunnel segment joint gasket is tested by carrying out the design indoor waterproof test. The test results are compared with the simulation results of the joint gasket, which verifies the accuracy of the theoretical analysis and numerical analysis conclusions in this paper. The specific conclusions are as follows:

1. According to the contact pressure formula, the waterproof performance of the sealing pad is affected by the height of the segment joint and the size of the joint opening. Increasing the offset angle of the structural joints towards each other can increase the sealing resistance of the upper sealing pad while reducing the sealing resistance of the lower sealing pad. Therefore, the offset angle of the structural joints towards each other has a significant impact on the waterproof performance of the sealing pad.
2. The stress analysis of the contact surface of the segment seal pad in the three-dimensional model shows that the impact of the step on the longitudinal left joint position is significant, and the tighter the sealing pad is squeezed, the greater the impact of the step on the longitudinal contact stress. However, when the contact area of the sealing pad is small, the impact of the opening on the longitudinal right contact stress is greater. Overall, the impact of the opening is greater than that of the step.
3. Comparing the numerical simulation model calculation with simulated waterproofing tests using sealing pads shows that the longitudinal left joint of the segment seal pad has relatively poor water resistance, indicating that when there is misalignment between segment joints, the waterproof performance of the segment seal pad at the higher-height joint is relatively weak. Additionally, under modified conditions, the waterproof rate of segment seal pad cross-sectional joints is greater than 40%, and the numerical simulation calculation is consistent with the waterproofing test results.

## Data Availability

All data generated or analysed during this study are included in this published article.
